# The Addition of *Opuntia ficus-indica* Ethanolic Extract to a Skimmed Milk-Based Extender Impacts Ram Sperm Quality

**DOI:** 10.1155/2023/6248890

**Published:** 2023-04-06

**Authors:** Larbi Allai, Xavier Druart, Pinar Terzioğlu, Noureddine Louanjli, Boubker Nasser, Mehmet Ozturk, Bouchra El Amiri

**Affiliations:** ^1^Laboratoire de Recherche Management de L'agriculture Durable (MAD), Ecole Supérieure de Technologie Sidi Bennour, Université Chouaïb Doukkali, El Jadida, Morocco; ^2^Animal Production Unit, Regional Center for Agricultural Research of Settat, National Institute for Agricultural Research (INRA), Avenue Ennasr, P.O. Box 415 Rabat Principal, 10090 Rabat, Morocco; ^3^Laboratoire de Biochimie et Neurosciences, Faculté des Sciences et Techniques, Université Hassan 1, BP 577, Setta 26000, Morocco; ^4^INRA, UMR 85 Physiologie de la Reproduction et des Comportements, Nouzilly F-37380, France; ^5^Faculty of Engineering and Natural Sciences, Department of Polymer Materials Engineering, Bursa Technical University, Bursa, Turkey; ^6^Labomac IVF Centers and Clinical Laboratory Medicine, Rue Moulay Abdellah N40, Casablanca, Morocco; ^7^Department of Chemistry, Faculty of Sciences, Mugla Sıtkı Koçman University, Mugla 48121, Turkey; ^8^African Sustainable Agriculture Research Institute (ASARI), Mohammed VI Polytechnic University (UM6P), Laayoune 70000, Morocco

## Abstract

Recently, researchers have focused on the use of natural antioxidants to improve semen quality as a key element for successful artificial insemination. In this context, the first aim of this study was to determine the antioxidant activity and composition (minerals, vitamins, and sugars) of *Opuntia ficus-indica* cladode ethanolic extract (ETHEX). A further purpose of the study was to investigate the effect of ETHEX supplementation on the quality of liquid ram semen extended with skim milk (SM) at 5°C. The antioxidant activity of ETHEX was studied using free radical 1, 1-diphenyl-2-picrylhydrazyl (DPPH•) assay. The mineral composition and the sugar and vitamin contents of ETHEX were determined using an inductively coupled plasma optical emission spectrometry (ICP-OES) and HPLC-DAD-RID analytical instruments. As a second part, semen was collected from five Boujaâd rams with an artificial vagina. The ejaculates with more than 70% motility were pooled, extended with skim milk (SM) extender without (control) or supplemented with 1–8% of ETHEX (37°C; 0.8 × 109 sperm/mL). Sperm quality parameters were assessed at 8, 24, 48, and 72 h. The results showed that ETHEX had a higher antioxidant activity compared to those of ascorbic acid and butylated hydroxytoluene (BHT). Furthermore, ETHEX contains a considerable amount of minerals, vitamins, and sugars. The inclusion of 1 or 2% ETHEX in SM increased the sperm motility, viability, and membrane integrity and decreased the abnormality of spontaneous and catalyzed lipids peroxidation (*p* < 0.05) up to 72 h. In addition, semen diluted with 1 and 2% ETHEX decreased the level of DNA fragmentation compared to the control group (*p* < 0.05). In conclusion, the ETHEX could be recommended to improve the quality of liquid ram spermatozoa. However, its effects on artificial insemination should be further studied.

## 1. Introduction

Artificial insemination with cryopreserved rams is not common in the sheep industry [1] because cryopreservation leads to a decrease in fertility. Acceptable fertility results have only been obtained with laparoscopic surgery. In fact, the cryopreservation process extremely decreased all sperm parameters compared to the liquid storage. Besides, semen cryopreservation is an expensive process, and for all cited reasons, liquid-stored semen can be an alternative to frozen-thawed semen for artificial insemination [2]. Therefore, some studies have motivated the use of liquid semen preservation for different species [3, 4]. One of the main factors associated with low sperm quality during liquid storage and cryopreservation is the production of reactive oxygen species (ROS), which occurs as an abnormal consequence of spermatozoa metabolism [5]. To prevent such stress, the semen needs to be diluted with suitable extenders and conditions. However, the addition of extenders significantly reduces the antioxidant capacity of spermatozoa and seminal plasma. The spermatozoa and seminal plasma system comprise taurine, reduced glutathione (GSH), glutathione peroxidase (GSH-Px), catalase (CAT), and superoxide dismutase (SOD) to prevent oxidative damage. The antioxidant system of spermatozoa is compromised during semen processing [6]. For that reason, the need for extender supplementation becomes necessary. Many additives with antioxidative properties have been reported to reduce the impact of ROS-induced and cold shock damage on sperm cells [7] and thus improve sperm ram quality [8–10].

Research has shown that plant extracts can be effective in improving semen quality in many animal species [11–14]. One of the key properties of plant extracts is their antioxidants activity, which is responsible for their ability to scavenge free radicals [15]. *Opuntia ficus-indica,* commonly known as the cactus, is a plant cultivated in many parts of the world, including North and South America, as well as Mediterranean countries such as Morocco and South Africa [16]. The ethanol extract from *Opuntia ficus-indica* cladodes is a natural source of antioxidants, which makes it effective in scavenging free radicals. This extract contains various constituents, such as tocopherol, polyphenols, flavonoids, quercetin, phenolic acids (caffeic and gallic acid), minerals, and sulfur amino acids (taurine, methionine, arginine, and cysteine) [17, 18]. Several studies have reported that this extract has anti-inflammatory and antioxidant properties [19].

*Opuntia ficus-indica* extract has been found to be beneficial for improving semen quality. Its antioxidant properties help to reduce oxidative stress, which is known to have a negative impact on sperm quality [20, 21]. Therefore, the use of *Opuntia ficus-indica* extract may be a natural and effective approach to improving semen quality in animals. *Opuntia ficus-indica* is a plant that contains a range of compounds, including polyphenols, flavonoids, quercetin, phenolic acids, and sulfur amino acids [22]. Both acetone and ethanolic extraction methods are commonly used to extract these compounds from the plant [23]. However, the choice of extraction method may depend on the specific compounds of interest. Acetone extraction is more effective in extracting nonpolar compounds such as lipids and pigments, while ethanolic extraction is better suited for polar compounds such as polyphenols and flavonoids due to its ability to extract a wider range of polar compounds. In a previous study, we utilized acetone extraction method to improve ram sperm quality [24], whereas in the current study, we are using ethanolic extraction to extract compounds from *Opuntia ficus-indica.* To the best of our knowledge, this is the first report focusing on the effect of *Opuntia ficus-indica* cladode ethanolic extract (ETHEX) on Boujaâd ram sperm quality parameters. The objective of this study was to determine the antioxidant properties, mineral, vitamin, and sugar composition of ETHEX and to assess its inclusion in SM on sperm total and progressive motility, viability, abnormality, membrane integrity, lipid peroxidation levels, and DNA fragmentation during liquid storage at 5°C up to 72 h.

## 2. Materials and Methods

### 2.1. Ethical Approval for Animal Study

Animal studies were conducted following the protocols of Animal Use and Care of the University of Hassan 1^st^, Settat, Morocco.

### 2.2. Chemicals and Reagents

All chemicals were purchased from Sigma–Aldrich (St. Louis, MO, USA) and Merck (Merck, Schuchardt, OHG, Germany). Ultrapure water was obtained from a Millipore Milli-Q system (Bedford, MA, USA).

### 2.3. Cactus Extraction and DPPH Inhibition Method

Nopal cactus cladodes were collected from the experimental station of Regional Center-INRA Settat, Morocco. They were washed with distilled water, dried at 55°C in an oven, and mechanically milled. The obtained powder was stored in a closed container at room temperature until use. To prepare the ethanol extract, a fine dried powder (5 g) was extracted by stirring at room temperature with 100 mL of ethanol/water mixture (70 : 30 v/v) for 4 days at 4°C [25]. After the solution was filtered, then the solvent was evaporated using a vacuum rotary evaporator (Buchi R-210) at 38°C. The remaining part was lyophilized and stored at 4°C until used.

Radical scavenging activity (RSA) of ETHEX was measured using the free radical 1,1- diphenyl-2-picrylhydrazyl (DPPH) [26]. A known amount of the dry ETHEX (0.1 mL) was centrifuged in methanol (2.9 mL) at 5000 rpm for 15 minutes followed by filtration through filter paper (Whatman No. 1). Methanolic solution of DPPH (0.5 mL, 100 *μ*M) was added to the tubes including the supernatant of each concentration. After vigorous shaking of the tubes, they were incubated at room temperature for 45 minutes in the darkness. Ascorbic acid and BHT were used as reference compounds. The lower absorbance of the reaction mixture indicated a higher free radical scavenging activity.

The percentage of free radical scavenging activities of the samples was calculated using the following formula:

(1) $$\begin{array}{c} \%RSA=\left[\left(A_{D}-A_{E}\right)/A_{D}\right]*100\text{,} \end{array}$$

where *A*_*D*_ and *A*_*E*_ is the absorbance of the DPPH blank sample and the absorbance of the test solution, respectively. *A*_*E*_ was the difference between the absorbance value of the test solution and its blank.

### 2.4. Total Sugar Content

The sample (2 g) was extracted with ultrapure water (20 mL) in an ultrasonic bath (Elmasonic, Germany) for 10 minutes at room temperature. Then it was filtered through a 0.45 µm PTFE filter. The total sugar content of the sample was determined using a high-performance liquid chromatography (HPLC) system (Agilent Technologies, USA) equipped with a refractive index detector (RID). The sugars were separated by NH_2_ column (Inertsil, 5 *μ*m, 4.6 mm × 250 mm, GL Sciences) maintained at 30°C. The injection volume was 10 *μ*L. The mobile phase was acetonitrile (80%) with a flow rate of 1.0 mL·min^−1^. Standard solutions were injected to get the retention time and standard curve for each sugar. The results were evaluated with the ChemStation Software and expressed as mg·g^−1^ dry weight.

### 2.5. Vitamin Content

The sample (1 g) was extracted with ultrapure water (10 mL) in an ultrasonic bath (Elmasonic, Germany) for 10 minutes at room temperature. After, 1 mL of 2 M NaOH and 12.5 mL of 1 M phosphate buffer (pH = 5.5) were added and fulfilled to 25 mL with ultrapure water. Then, the sample was filtered through a 0.45 µm PTFE filter before injection. The vitamin content of the sample was determined using a high-performance liquid chromatography (HPLC) system (Agilent Technologies, USA) equipped with a diode array detector (DAD). The vitamins were separated on an ODS column (5 *μ*m, 4.6 mm × 250 mm, Inertsil, GL Sciences) maintained at 30°C. The injection volume was 10 *μ*L. The mobile phases were the aqueous solution of trifluoroacetic acid (0.025%, v/v), solution A; and acetonitrile, solution B. The elution program was the gradients of solvent B as following: 0.0% solvent B (0–5^th^ min), 25% solvent B (6−11^th^ minutes), 45% solvent B (11−19^th^ minutes), 40% solvent B (19−20^th^ minutes), 0% solvent B (20−22^nd^ minutes). Standard solutions of vitamins (Vitamins C, B2, B3, B5, and B9) were injected to get the retention time and standard curve for each vitamin. The results were evaluated with the ChemStation Software and expressed as mg/100 g dry weight.

### 2.6. Mineral Content

The digestion of the sample was performed on a microwave digestion system (CEM MARS5 (USA)). Approximately 0.5 g of dry sample was transferred to a PTFE digestion tube containing 6 mL of nitric acid (65%) and 2 mL of hydrogen peroxide. The operating conditions of the microwave oven were as follows temperature (150–200°C), ramp (20 min), time (2 min), and power (100%) for each step. The digested sample was cooled to room temperature, filtered, and the filtrate was diluted by adding 100 mL of ultrapure water. The mineral concentration was determined by the inductively coupled plasma optical emission spectrometry (ICP-OES), (Agilent 5100, USA). The nitrogen content was estimated by the Kjeldahl method [27].

### 2.7. Animals, Semen Collection, and Processing

Five Boujaâd mature rams (weight: 80–85 kg, aged between 3 and 4 years) were used as semen donors. They were maintained at the station of animal reproduction biology at the Research Regional Center, Settat INRA-Morocco. A total number of 50 ejaculates were collected from the rams using an artificial vagina, during the breeding season (July to September) and then the semen was pooled to minimize individual variation. After semen collection, the semen (10 *µ*L) was placed on a glass slide without a coverslip, and the wave motion of semen was evaluated (0–5 scale) after judging five different microscopic fields. The sperm concentration was determined by a spectrophotometer. Ejaculates, which met the following criteria: the volume of 0.5–2 mL; good wave motion (≥3 on a 0–5 scale); ≥2.5 × 109 spermatozoa/mL; and ≥70% motile sperm were evaluated and used for the next step. All ejaculates were pooled to eliminate individual differences and diluted with skim milk (SM) as the base extender. It was prepared by diluting skim milk (11 g) in distilled water (100 mL) and then heated at 95°C for 10 minutes. Penicillin and streptomycin (0.05 mg/mL) were added to the extender. The semen was diluted with the base extenders at 37°C, containing 0 (control), 1, 2, 4, and 8% of ETHEX to reach a final concentration (0.8 × 109 sperm/mL) (single step dilution).

The semen samples were then cooled from 37 to 5°C. Sperm total motility, progressive motility, viability, abnormality, membrane integrity, and lipid peroxidation were determined at 8, 24, 48, and 72 h. The combination of extender × antioxidant concentration giving the best protective effects on sperm progressive motility was selected to assess DNA fragmentation at 8, 24, 48, and 72 h using the tunnel technique.

### 2.8. Evaluation of Semen Characteristics

Sperm total and progressive motility were assessed by a computer-assisted sperm analysis system (ISAS, version 1.0.17, Proiser, Valencia, Spain) by analyzing five fields by sample (200 sperms/field). The semen was diluted with PBS-BSA to reach 20 × 106 sperm/mL at 37°C. The sperm motility was assessed using a 10× negative phase contrast objective on a UB203 microscope (UOP/Proiser, Paterna, Valencia, Spain). Spermatozoa were defined as nonmotile if average path velocity (VAP) was lower than 10 *µ*m/s and sperm cells were considered as progressive motile when VAP was higher than 75 µm/s and straightness index (STR) was higher than 80%.

For viability assessment, eosin-Y (1.67 g), nigrosin (10 g) and sodium citrate (2.9 g) were dissolved in distilled water (100 mL), and then nigrosin–eosin stain (10 *µ*L), and sperm dilution (5 *µ*L) were mixed on a glass slide [28]. The mixture was smeared and examined with bright-field microscopy (400x). A total number of 200 spermatozoa were counted. Sperm showing partial or complete purple heads were considered nonviable or dead, and only sperm showing strict exclusion of the stain were considered alive.

The hypoosmotic swelling test (HOST) was used to evaluate the functional integrity of the sperm plasma membrane [29]. The solution for the HOST assay consisted of fructose (9 g) and sodium citrate (4.9 g) per liter of distilled water. For sperm tail assessment, semen (30 *µ*L) was mixed with hypoosmotic solution (300 *µ*L, 100 mOsM) and incubated for 50–60 minutes at 37°C. After incubation, 10 *µ*L of the mixture was spread with a coverslip on a warm slide. A total number of 200 spermatozoa were counted at least using five different microscopic fields at 400x. The percentage of spermatozoa with swollen and curved tails was recorded.

The morphology of sperm was evaluated using the Diff-Quik kit (Diagnostic Systems S.L. Barcelona, Spain). Briefly, 3 *µ*L of diluted semen was smeared on a glass slide and allowed to air-dry. The slide was then dipped into a fixative solution for 1 min and the first and second solutions seven to ten times. Between the fixing step and each of the staining steps, the excess solutions were dried from the slides by placing them vertically on absorbent paper. At least 200 sperms were evaluated under light microscopy at 1000x magnification using UB203 microscope (UOP/Proiser, Paterna, Valencia, Spain).

### 2.9. Measurement of Lipid Peroxidation

Malondialdehyde (MDA) concentrations, as indices of lipid peroxidation (LPO) in the semen samples, were measured using the thiobarbituric acid reaction [30]. The thiobarbituric acid reactive substances (TBARS) were measured in the semen spontaneous LPO or after incubation with 0.24 mM of FeSO4 at 37°C in a water bath for 60 minutes (iron-catalyzed LPO). The TBARS concentration was determined by comparing the sample's absorbance at 532 nm with a standard curve prepared using MDA. The results were expressed in nmol TBARS/10^8^ sperm.

### 2.10. Assessment of DNA Fragmentation

For the terminal deoxynucleotidyl transferase dUTP nick end labeling (TUNEL) technique, the *In-Situ* Cell Death Detection Kit with fluorescein (Roche Diagnostics GmbH, Mannheim, Germany) has been used according to Nur et al. [31]. Briefly, different aliquots of semen samples were diluted with phosphate-buffered saline (PBS) and centrifuged at 400 × *g* for 10 minutes. One drop of resuspended spermatozoa was smeared on a glass slide and fixed with 10% formaldehyde for 30 minutes at room temperature.

The slides were washed three times with PBS (5 minutes each), treated in a humidified chamber with proteinase K for 10 minutes, washed with PBS, treated with 3% H_2_O_2_ in distilled water for 10 minutes at room temperature, and washed again with PBS. The slide was permeabilized with 0.1% Triton X-100 for 5 minutes on ice. The slides were incubated in dark at 37°C for one hour with the TUNEL reaction mixture. After labeling, samples were washed with PBS and analyzed immediately using fluorescence microscopy (Zeiss Eurostar, Germany 100×). The percentage of TUNEL-positive sperm was determined by the evaluation of at least 100 sperm.

### 2.11. Statistical Methods

The data were analyzed statistically using JMP15.0 (SAS Institute Inc., Cory, NC, USA) program. All dependent variables were submitted to the Shapiro–Wilk test normality and homogeneity analysis. Dependent variables with normal distribution were evaluated using variance analysis with the comparison of means using Tukey's test. The results were expressed as the mean ± SEM (standard error meaning). The differences with values of *p* < 0.05 were considered to be statistically significant.

## 3. Results

### 3.1. Radical Scavenging Activity

The scavenging ability of antioxidant substances was evaluated by the DPPH assay. The results of the scavenging activities of ETHEX, ascorbic acid, and BHT are presented in Table 1. The results indicate that the antioxidant activity of the ETHEX was higher than that of ascorbic acid and BHT in all tested concentrations.

### 3.2. Sugar, Vitamin, and Mineral Contents

As shown in Tables 2–4, the major sugars presented in ETHEX were saccharose (2.85 mg·g^−1^) and glucose (2.67 mg·g^−1^). The fructose content was found to be 0.56 mg·g^−1^ (Table 2). As seen in Table 3, vitamins C, B2, B9, and B3 were detected in ETHEX. Among them, the major vitamin was B2 (1384 ± 5.04 mg/100 g), followed by vitamin C (963.1 ± 3.04 mg/100 g), and vitamin B3 (91.79 ± 0.19 mg/100 g). The vitamin B5 was undetected in the ETHEX.

The mineral content of ETHEX (Table 4) revealed the presence of boron, calcium, copper, iron, manganese, magnesium, nitrogen, phosphorus, potassium, sodium, and zinc. The copper (0.004 g/100 g); manganese (0.005 g/100 g); zinc (0.003 g/100 g); and boron (0.003 g/100 g) concentrations were found to be lower. The most abundant mineral was potassium followed by nitrogen, calcium, and magnesium. The data showed that ETHEX contains iron (0.020 g/100 g).

### 3.3. Microscopic Sperm Parameters

**Figure 1:**
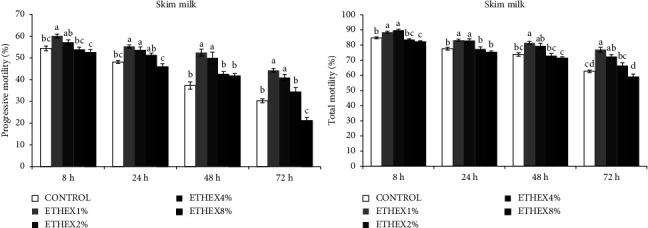
Effect of ETHEX on sperm motility during liquid storage in a SM extender. Mean ± SEM values of sperm motility of ram semen stored for 8–72 h at 5°C in SM extender supplemented with ETHEX 1–8% w/v. Different superscripts indicate a significant difference between ETHEX concentrations within each duration of storage (*p* < 0.05).

**Figure 2:**
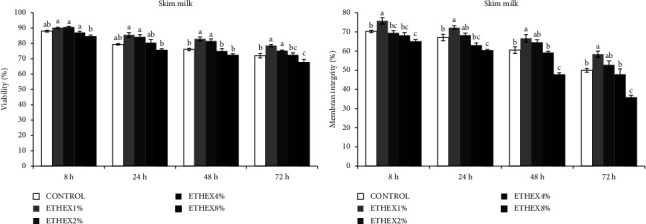
Effect of ETHEX on viability and membrane integrity during liquid storage in a SM extender. Mean ± SEM values of viability and membrane integrity of ram semen stored for 8–72 h at 5°C in SM extender supplemented with ETHEX 1–8% w/v. Different superscripts indicate a significant difference between ETHEX concentrations within each duration of storage (*p* < 0.05).

**Figure 3:**
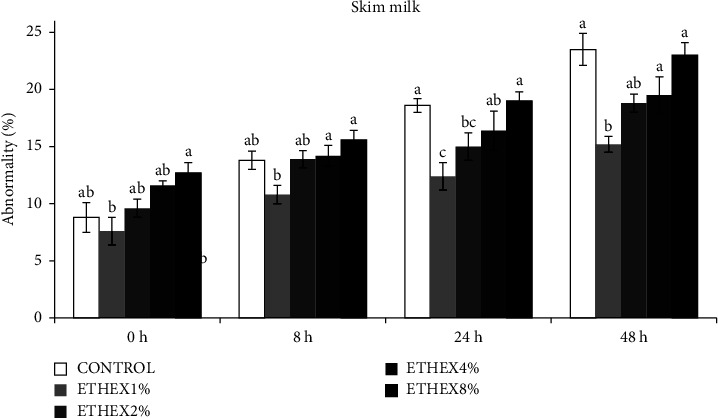
Effect of ETHEX on abnormality during liquid storage in SM extender. Mean ± SEM values of abnormality of ram semen stored for 8–72 h at 5°C in a SM extender supplemented with ETHEX 1–8% w/v. Different superscripts indicate a significant difference between ETHEX concentrations within each duration of storage (*p* < 0.05).

The effects of ETHEX on sperm motility, viability, membrane integrity, and abnormality of ram semen during different storage times at 5°C are presented in Figures 1–3.

The effect of ETHEX on sperm total motility and progressive motility was significantly higher in SM supplemented with 1 and 2% compared to the control groups (Figure 1). However, no difference was found between SM with 4% ETHEX and the control group (Figure 1).

Regarding viability, a significant difference was recorded between the control and the SM with 1 and 2% ETHEX (*p* < 0.05) after 48 h of storage (Figure 2). In contrast, no difference was found between the control and other samples (SM with 4% and 8% ETHEX) (Figure 2). Whereas the enrichment of the basic extender with 1% ETHEX resulted in a higher percentage of membrane integrity compared to the control groups (Figure 2).

The results for abnormalities up to 72 h of incubation with different concentrations of ETHEX in SM are presented in Figure 3. These results showed that the addition of 1% of ETHEX to SM decreases the percentage of abnormality compared to the control (*p* < 0.05).

### 3.4. Malondialdehyde Concentration

The spontaneous and catalyzed lipid peroxidation levels in ram semen containing different concentrations of ETHEX for different storage periods at 5°C are given in Table 5. The level of spontaneous and catalyzed LPO in control groups was significantly higher when compared to the experimental groups supplemented with 1 and 2% of ETHEX during the storage period at 5°C in SM. The skim milk with 1% of ETHEX maintained a better effect when compared to the other concentrations. While no effect (*p* < 0.05) was observed when the concentration exceeded 2% compared to the control group.

### 3.5. DNA Fragmentation Levels

The effect of ETHEX on DNA fragmentation of liquid-stored ram semen at different periods is shown in Table 6. It was determined that using the selected concentrations was statistically significant in sperm DNA damage of ram semen during liquid storage. The DNA fragmentation was significantly reduced, in comparison to the controls, with the addition of 1% ETHEX after 8 h of storage and 2% ETHEX to SM after 24 and 72 h of storage.

## 4. Discussion

To overcome the loss of semen quality during storage, several extracts from plants have been listed as natural antioxidants that could target such effects [32–34]. Even, to the best of our knowledge, there is no report in the literature on the supplementation of ram semen with ETHEX. Thus, the present study was carried out to test the ability of ETHEX at four concentrations (1, 2, 4, and 8%) to prevent the harmful effect of handling liquid ram semen in SM at 5°C up to 72 h. The main finding that emerged from this study was that the beneficial influence of ETHEX resulted in higher overall quality of ram semen. The inclusion of 1 and 2% ETHEX to the ram semen extended in SM at 5°C increases sperm motility, viability, and membrane integrity and decrease sperm abnormality. In contrast, concentrations above this threshold (2%) showed no significant results on sperm quality.

The beneficial effects recorded while using 1% and 2% ETHEX are probably due to synergistic effects of multiple constituents of natural extracts and not to the single purified active compounds [35]. The ETHEX characterization showed a considerable radical scavenging activity exceeding that of BHT and ascorbic acid (Table 1). Besides, the beneficial effects of ETHEX on lipid peroxidation and DNA fragmentation were also recorded.

Numerous researchers have already pointed out that ETHEX contains an important antioxidant compound ranging from flavonoids to phenols [36]. Some of them have the potential to act as scavengers, superoxidase, and hydroxyl and peroxy radicals released from oxidative phosphorylation. Together they can avoid the free radical's formation and protects the mitochondria, DNA, and plasma membrane of sperm [37–39]. Prevent oxidative damage could be achieved by a variety of different mechanisms, such as free radical scavenging, transition metal chelation and interactions with lipid membranes, proteins and nucleic acids [40]. In addition to the previously mentioned compounds, ETHEX contains sugars (glucose, fructose, and sucrose) and vitamins (C, B2, B3, and B9). The inclusion of such compounds in extenders has been shown to improve semen quality [41, 42]. More precisely, sugars and vitamins have beneficial antioxidative and protective effects against the cold shock and freeze-thaw damage of sperm [43–46]. Sugars act as plasma membrane protectors and increase the integrity percentage in several animals' species [47, 48]. Furthermore, the sugars provide the main energy source required for metabolic processes during spermatozoa development [49]. Regarding the vitamins, an improvement of ram semen quality observed in the current study could be attributed to the presence of Vitamin C. Previous studies stated the improvement of sperm viability and motility and a decrease in the percentage of abnormality [50, 51]. Coming back to minerals, the characterization of the ETHEX revealed that it contained calcium, magnesium, sodium, and zinc (Table 4). Especially, zinc has been reported to protect spermatozoa against oxidative stress and subsequently improve male fertility [52]. Furthermore, Zn showed a positive outcome regarding antioxidant activity [53, 54]. It plays an important role in controlling motility by controlling the use of energy through adenosine triphosphate systems and by regulating the energy stores of phospholipids [55]. Additionally, to zinc, calcium triggers the acrosomal reaction in mammalian spermatozoa and is also involved in sperm motility [56] and inhibits the enzyme phosphodiesterase, which prevents cAMP degradation and enhances sperm motility [57].

## 5. Conclusion

In conclusion, the findings of the present study imply that supplementation of SM with ETHEX at 1 and 2% level has a beneficial effect on quality parameters of liquid ram semen stored up to 72 h, which may be due to the antioxidant effects of one or more active compounds presented in ETHEX. However, additional studies are required to reveal the active components existing in ETHEX and test the impacts of this extract's inclusion in the freezing media and the success of AI in sheep.

## Figures and Tables

**Table 1:** Radical scavenging activity (inhibition %) of ETHEX, ascorbic acid and BHT, by the DPPH method.

Concentration (*µ*g/ml)	ETHEX	Ascorbic acid	BHT
0.5	40.10	34.03	24.69
1	46.50	38.78	32.10
4	60.00	59.99	54.35
8	82.10	79.26	70.27
16	97.66	90.80	77.10
32	99.85	93.35	91.23

**Table 2:** Sugar composition of ETHEX^a^.

Sugars	Concentration (mg/g)
Sucrose	2.85 ± 0.02
Glucose	2.67 ± 0.01
Fructose	0.56 ± 0.001

^a^The results were the average of three parallel measurements ± SEM (*p* < 0.05).

**Table 3:** Vitamin composition of ETHEX^a^.

Vitamins	Concentration (mg/100 g)
C	963.1 ± 3.04
B2	1384 ± 5.04
B9	12.18 ± 0.11
B3	91.79 ± 0.19
B5	—^b^

^a^The results were the average of three parallel measurements ± SEM (*p* < 0.05). ^b^not detected.

**Table 4:** Mineral composition of ETHEX^a^.

Minerals	Concentration (g/100 g)
Nitrogen	2.49 ± 0.04
Phosphorus	0.35 ± 0.004
Potassium	4.55 ± 0.06
Calcium	1.93 ± 0.03
Magnesium	1.25 ± 0.02
Iron	0.020 ± 0.001
Copper	0.004 ± 0.001
Manganese	0.005 ± 0.001
Zinc	0.003 ± 0.001
Boron	0.003 ± 0.001
Sodium	0.724 ± 0.001

^a^The results were the average of three parallel measurements ± SEM (*p* < 0.05).

**Table 5:** The effect of ETHEX on lipid peroxidation after 8, 24, 48 and 72 hours of ram liquid semen stored at 5°C in SM.

	Groups	*Storage time (hours)*			
		8	24	48	72
SLPO	CONTROL	0.40 ± 0.02^bc^	0.89 ± 0.04^a^	1.75 ± 0.03^a^	2.34 ± 0.03^ab^
	ETHEX 1%	0.28 ± 0.02^c^	0.38 ± 0.02^b^	0.86 ± 0.04^d^	1.35 ± 0.09^d^
	ETHEX 2%	0.47 ± 0.05^b^	0.80 ± 0.05^a^	1.33 ± 0.09^c^	1.63 ± 0.03^c^
	ETHEX 4%	0.55 ± 0.07^ab^	0.84 ± 0.05^a^	1.54 ± 0.06^bc^	2.13 ± 0.05^b^
	ETHEX 8%	0.72 ± 0.03^a^	0.88 ± 0.05^a^	1.67 ± 0.04^ab^	2.44 ± 0.07^a^

ILPO	CONTROL	1.56 ± 0.12^a^	1.66 ± 0.06^a^	2.04 ± 0.03^a^	3.40 ± 0.10^a^
	ETHEX 1%	0.64 ± 0.03^c^	0.75 ± 0.06^c^	1.52 ± 0.08^b^	1.78 ± 0.09^c^
	ETHEX 2%	0.68 ± 0.05^c^	1.33 ± 0.07^b^	1.79 ± 0.06^ab^	2.47 ± 0.08^b^
	ETHEX 4%	1.08 ± 0.07^b^	1.35 ± 0.06^b^	2.03 ± 0.07^a^	3.20 ± 0.06^a^
	ETHEX 8%	1.05 ± 0.08^b^	1.63 ± 0.04^a^	2.06 ± 0.07^a^	3.41 ± 0.08^a^

SLPO: spontaneous LPO; ILPO: induced LPO. Values are expressed as mean ± SEM. Different superscripts^(a,b)^ within the same column indicate a significant effect of the extender within each duration of storage.

**Table 6:** The effect of ETHEX on DNA fragmentation index after 8, 24, 48 and 72 hours of ram liquid semen stored at 5°C in SM.

Group	*Storage time (h)*			
	8	24	48	72
CONTROL	4.70 ± 0.48^a^	9.75 ± 1.11^a^	14.5 ± 0.29^a^	15.75 ± 0.63^a^
ETHEX 1%	1.75 ± 0.41^b^	3.50 ± 0.29^b^	6.25 ± 0.25^b^	8.50 ± 0.50^b^
ETHEX 2%	3.50 ± 0.50^ab^	5.40 ± 0.63^b^	8.80 ± 0.45^b^	10.75 ± 0.80^b^

The DNA fragmentation index (%) was analyzed for semen stored during 72 hours at 5°C in skim milk supplemented with selected ETHEX concentrations. Values are expressed as mean ± SEM. Values are expressed as mean ± SEM. Different superscripts^(a,b)^ within the same column indicate a significant effect of the extender within each duration of storage.

## Data Availability

The data for this article are made available when needed.
